# *sy680* is a novel allele of *pkd-2*

**DOI:** 10.17912/W2SW9M

**Published:** 2017-09-28

**Authors:** Allyson Whittaker, Gary Schindelman, Shala Gharib, Paul W. Sternberg

**Affiliations:** 1 Division of Biology and Biological Engineering, California Institute of Technology, Pasadena, California, USA​; 2 Howard Hughes Medical Institute, California Institute of Technology, Pasadena, California, USA

**Figure 1.**
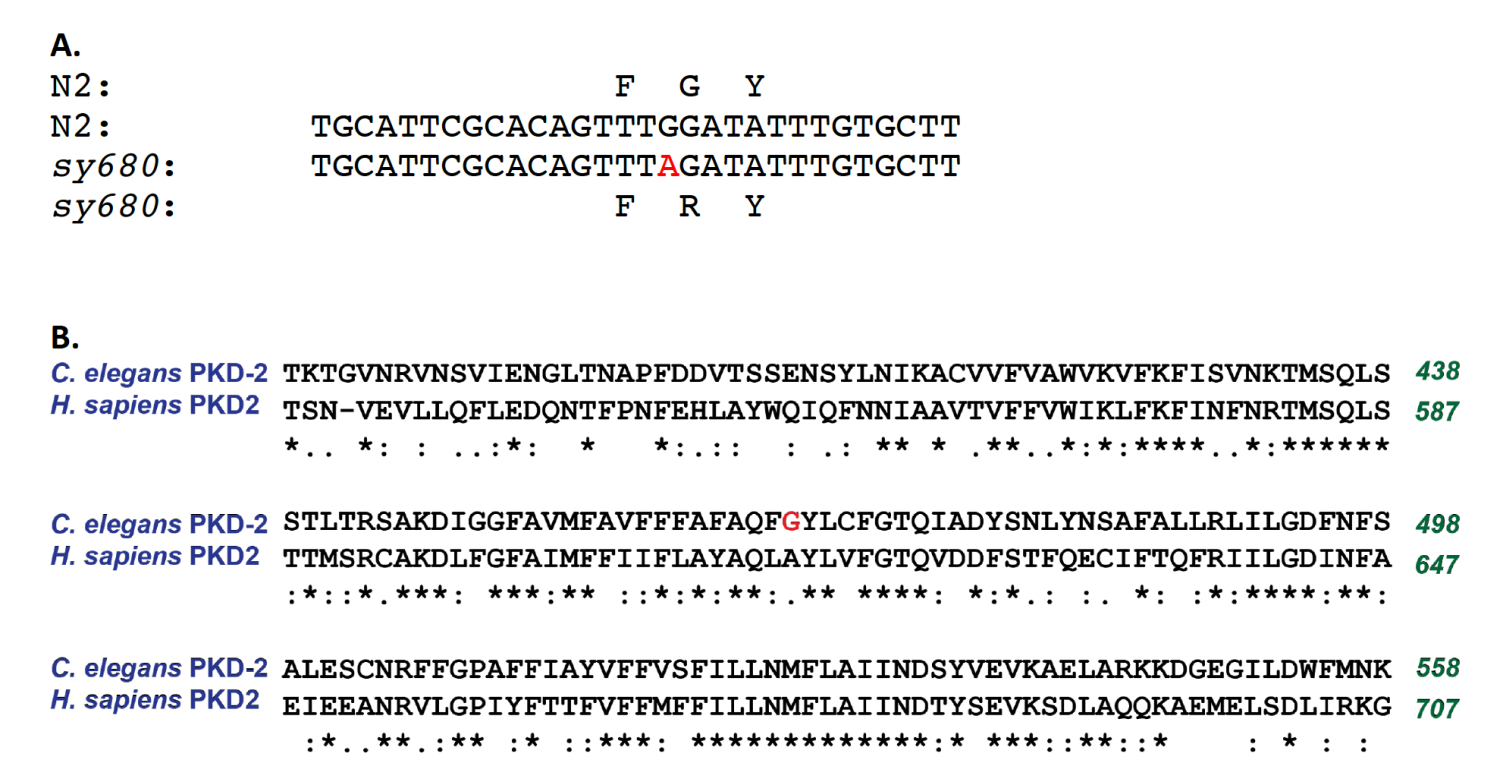
A. The position of lesion in the *pkd-2* DNA sequence. B. Location of lesion in an alignment of *C. elegans*and human proteins. Position of substitution shown in red.

## Description

**Summary** a new allele of *pkd-2* was isolated in a behavioral genetic screen for male mating defects, and found to result in a substitution of Arginine for Glycine in the equivalent of human PKD2 alanine 615.

**Article**The *C. elegans* ortholog of polcystin-2 is encoded by *pkd-2* (Barr et al., 2001). From an EMS screen of a *plg-1; him-5* strain for male mating defective mutants and a secondary behavioral screen for defects in discrete steps of male mating behavior, namely response to contact to hermaphrodites and vulval location (described in Schindelman et al., 2006), we identified a new allele of *pkd-2* based on mapping and complementation. *sy680*fails to complement *pkd-2**(**sy606**)* for defects in response to contact with hermaphrodite and vulval location. Here we report the sequence of this allele. PCR amplification and sequencing of *pkd-2* exons indicated that there was a c–>t transition in the transcribed DNA strand (g–>a in the *pkd-2* sense strand; Figure 1A). This change leads to an altered codon, a Glycine to Arginine substitution the PKD-2 protein. This position corresponds to A615 of the human protein (Figure 1B).

## Reagents

**Strains:**PS7518 *plg-1**(e2001d) III;* *pkd-2**(sy680) IV;* *him-5**(**e1490**) V*

PS3400 *pkd-2**(**sy606**)*
